# Urinary Soluble CD163 Levels Predict IgA Nephropathy Remission Status

**DOI:** 10.3389/fimmu.2021.769802

**Published:** 2021-12-23

**Authors:** Shaomin Gong, Shi Jin, Yang Li, Wuhua Jiang, Zhen Zhang, Ziyan Shen, Jialin Wang, Huili Zhou, Xiao Liu, Xialian Xu, Xiaoqiang Ding, Yiqin Shi, Hong Liu

**Affiliations:** ^1^ Department of Nephrology, Zhongshan Hospital, Fudan University, Shanghai, China; ^2^ Shanghai Medical Center of Kidney Disease, Shanghai, China; ^3^ Shanghai Institute of Kidney and Dialysis, Shanghai, China; ^4^ Shanghai Key Laboratory of Kidney and Blood Purification, Shanghai, China; ^5^ Hemodialysis Quality Control Center of Shanghai, Shanghai, China

**Keywords:** biomarker, IgA nephropathy, urinary soluble CD163, remission status, macrophages

## Abstract

Noninvasive biomarkers of disease activity are needed to predict disease remission status in patients with IgA nephropathy (IgAN). Soluble CD163 (sCD163), shed by monocytes and macrophages, is a potential biomarker in diseases associated with excessive macrophage activation. We investigated the association of urinary sCD163 (u-sCD163) with histopathological activity and clinical manifestations in 349 patients with biopsy-diagnosed IgAN. U-sCD163 was measured *via* enzyme-linked immunosorbent assay. In patients with IgAN, higher u-sCD163 levels were associated with histological lesions of greater severity, as well as more proteinuria and poorer renal function. Additionally, u-sCD163 was correlated with infiltration of tubulointerstitial CD163^+^ macrophages. High u-sCD163 levels (>3.57 ng/mg Cr) were associated with a 2.66-fold greater risk for IgAN remission failure in adjusted analyses. Adding u-sCD163 levels to the model containing clinical data at biopsy and MEST-C score significantly improved the risk prediction of IgAN remission status (AUC 0.788). Together, our results suggest that u-sCD163 may be a useful noninvasive biomarker to evaluate disease severity and remission status of IgAN.

## Introduction

IgA nephropathy (IgAN) is the most common form of primary glomerulonephritis throughout the world and is a leading cause of chronical kidney disease (CKD) and kidney failure (1). Approximately 20–40% of IgAN patients develop end-stage kidney disease (ESKD) within 20 years from the time of diagnosis (2). Patients can present with a range of traits, from asymptomatic microscopic haematuria to macroscopic haematuria and/or proteinuria. Furthermore, no specific therapy is available for IgAN. So development of strategies for early diagnosis and precise treatment for IgAN are urgently needed. Currently, IgAN diagnosis is based on kidney biopsy, an invasive procedure. Risk of bleeding and other clinical complications limits the application of biopsy. Therefore, noninvasive and sensitive biomarkers are necessary for evaluating disease severity in patients with IgAN.

Macrophages are involved in the development of multiple kidney diseases. In IgAN, macrophages are among the most important inflammatory cells, playing a key role in mediating the glomerular and interstitial injuries (3). It was implicated that Fc receptors (FcαRI or CD89) triggering the massive influx of macrophages into IgAN in a FcRγ-dependent manner, and was crucial for disease progression towards renal failure (4). Recently, a study of single-cell transcription revealed that macrophages are the main immune cells in the kidneys of IgAN patients, and the inflammatory cytokines and chemokines secreted by mesangial cells may contribute to macrophages recruitment to the renal interstitum (5). Macrophages can be broadly divided into two types: the classically activated macrophage (M1) and the alternatively activated macrophages (M2) (6). M2 macrophages can be further subdivided into three subgroups. Wound-healing macrophages (M2a), M2b and regulatory macrophages (M2c) (7). Macrophages infiltration in IgAN kidneys are predominantly composed of CD163^+^ M2c macrophages (8).

CD163 is a 130kDa transmembrane protein, a member of the cysteine-rich scavenger receptor superfamily type B originally described as a scavenger receptor for hemoglobin-haptoglobin complexes (9). It is a surface marker expressed by M2c macrophages that infiltrate tissues during the healing phase of inflammation (10). Soluble CD163 (sCD163) is derived from the cleavage of the CD163 macrophage receptor by metalloproteinases (11). Previous studies reported elevated urinary sCD163 (u-sCD163) levels in patients with active lupus nephritis (LN) and antibody-associated glomerulonephritis (ANCA-GN), suggesting that u-sCD163 is a potential biomarker of kidney inflammation in LN and ANCA-GN (12–14). However, no study has addressed u-sCD163 levels in IgAN.

Therefore, this study validated the association of urinary biomarker of CD163 with clinical and histological manifestations in IgAN patients. Because u-IL6 and urine monocyte chemoattractant protein-1 (u-MCP-1) are also predictive markers of disease outcome and treatment efficacy (15), we measured their concentrations in our IgAN cohort, then compared the performance in predicting IgAN remission across all three biomarkers. We examined whether adding biomarkers to clinical and histologic data at the time of biopsy would improve prediction performance. Our aim was to develop a noninvasive biomarker for disease severity assessment and prognosis prediction in patients with IgAN.

## Meterials and Methods

### Study Population

The study retrospectively examined 349 patients with biopsy-diagnosed IgAN. Diagnosis occurred from January 2018 to December 2019 in Zhongshan Hospital, Fudan University. Glomeruli count was required to be at least eight per biopsy section. Patients were excluded if they fit one or more of the following criteria: <18 years old, end-stage kidney disease (ESKD) on admission (estimated glomerular filtration rate [eGFRs] <15 mL/min/1.73 m^2^), secondary mesangial IgA deposition (e.g., hepatitis related glomerulonephritis, systemic lupus erythematosus, rheumatoid arthritis, Henoch Shönlein Purpura), urinary tract infection or septicemia at time of diagnosis, and use of steroids or immunosuppressants within 3 months before diagnosis.

**Figure 1:**
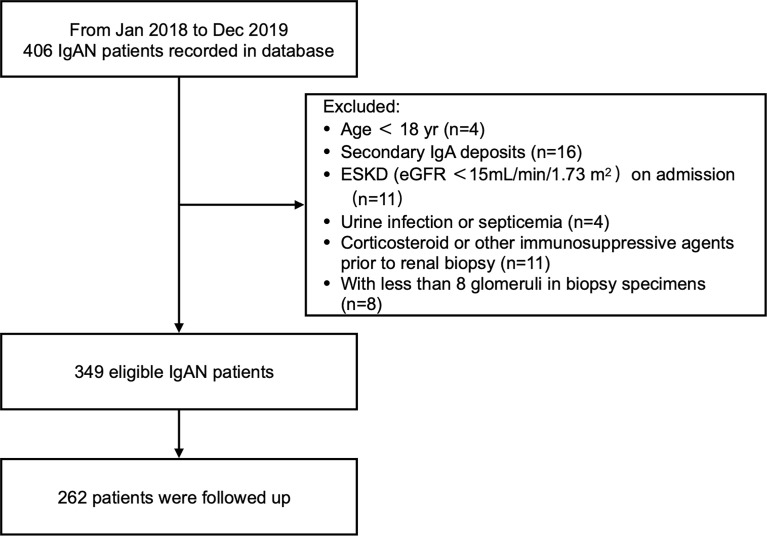
Flow chart of enrollment and exclusion.

At 6 months after initiation of therapy, 262 patients were followed up to evaluate treatment response (**Figure 1**). Procedures were reviewed and approved by the Ethics Committee of Zhongshan Hospital (B2021-027). All patients provided written informed consent.

### Samples

Urine from the first morning was collected on the day of kidney biopsy. Urine and blood samples were centrifuged for 10 min at 600 × g and 4°C. Supernatants were collected and frozen at -80°C until measurement. An experienced renal pathologist blinded to patient outcomes reviewed all kidney biopsy slides.

### Clinical and Pathologic Manifestations

Patient characteristics and laboratory measurement, including age, sex, body mass index (BMI), mean arterial pressure (MAP), hemoglobin, serum albumin, C-reactive protein (CRP), serum creatinine, 24-hour proteinuria, were collected retrospectively from medical records at the time of kidney biopsy. Hypertension was defined as systolic BP (SBP) of ≥140mmHg, diastolic BP of ≥90mmHg, or use of antihypertension drugs. The eGFR was calculated using the creatinine-based CKD Epidemiology Collaboration (CKD-EPI) equation (16).

Diagnosis of IgAN was based on optical microscopy and immunofluorescence. Evaluation of kidney histologic lesions were graded according to MEST-C score (17), which was defined as the sum of each histological score in Oxford Classification. Mesangial score was recorded as number of cells per mesangial area (M1 or M0). Endocapillary hypercellularity was scored as presence (E1) or absence (E0), as was segmental sclerosis (S0, S1). Interstitial fibrosis or tubular atrophy was classified based on area with tubulointerstitial damage: ≤25% (T0), 26% to 50% (T1), or >50% (T2). Crescent lesions were scored as C0 (no crescents), C1 (crescents in <25% of glomeruli), and C2 (crescents in ≥25% of glomeruli). Total number of glomeruli, globally sclerotic glomeruli, and glomeruli with segmental sclerosis were also counted. Global glomerulosclerosis (global sclerotic glomeruli/total glomeruli × 100%) and segmental sclerosis (segmental sclerotic glomeruli/total glomeruli × 100%) were recorded as percentages. A ‘tubulointerstitial score’ was used to assess tubulointerstitial damage according to tubular atrophy (0, none; 1, 1-25%; 2, 26-50%; 3, >50% of cortical area involvement), interstitial fibrosis (0, none; 1, 1-25%; 2, 26-50%; 3, >50% of cortical area involvement) and interstitial inflammation (0, none; 1, 1-25%; 2, 26-50%; 3, >50% of cortical area involvement) (18).

At 6 months after the initiation of therapy, remission status was assessed based on proteinuria, serum creatinine, and eGFR. Proteinuria and eGFR were determined at 6 months after kidney biopsy. Remission status was categorized into complete remission (CR), partial remission (PR) or remission failure (RF). Absence of proteinuria (proteinuria ≤ 0.3 g/24 h) and lack of worsening of kidney function (<30% reduction in eGFR from baseline) indicated CR. Proteinuria ≤ 1 g/24 h and lack of worsening of kidney function (<30% reduction in eGFR from baseline) indicated PR. Finally, RF was defined as urine protein > 1 g/24 h or proteinuria increase from baseline, worsening of kidney function (≥30% reduction in eGFR), or ESKD (19). ESKD was defined as eGFR<15mL/min per 1.73 m^2^ or need for renal replacement therapy (RRT; including hemodialysis, peritoneal dialysis, or renal transplantation). Renal RF at 6 is considered short-term RF (14).

### Immunohistochemical Analysis of Kidney Biopsy Specimens

Kidney biopsy specimens were fixed with 10% formalin, embedded in paraffin, and cut into 4 μm sections for immunohistochemistry. Sections were labelled with mouse anti-human CD163 monoclonal IgG1 (clone 10D6; Abcam, Cambridge, UK). Average number of CD163-positive cells in glomeruli and interstitium were determined at 400 × magnification. Glomerular infiltrate is expressed as number of positive cells/glomerulus. Interstitial infiltrate is expressed as numbers of positive cells/high power field (HPF). Five fields were selected.

### Measurement of Urinary Cytokines

U-sCD163, IL-6, and MCP-1 concentrations were measured using enzyme-linked immunosorbent assay (ELISA) kits following manufacturer protocol (sCD163: EUROIMMUN Medizinische Labordiagnostika AG; IL-6: DY206, R&D Systems; MCP-1: DY279, R&D Systems). Urinary biomarker levels were normalized using urinary creatinine and expressed as ng/g creatinine. All samples were processed in duplicate.

### Statistical Analysis

Statistical analyses were performed in SPSS 26.0. All tests were two-tailed, and significance was set at *P*<0.05. Variables with normal distribution were expressed as mean ± standard deviation (SD). Between-group differences were assessed using one-way analysis of variance (ANOVA) and independent-sample *t* test. Nonparametric variables u-sCD163/Cr, u-IL6/Cr, and u-MCP-1/Cr levels were log-transformed before being compared using ANOVA or *t* tests.

Biomarkers u-sCD163, u-IL6, and u-MCP-1 were categorized into three levels (low, middle, and high) using tertiles. Uni- and multivariate logistic models were used to examined the effect of biomarker levels on IgAN remission status. Adjusted odds ratio (OR) and 95% confidence interval (CI) were applied to quantify the association between biomarkers and IgAN remission status. Covariates included age, sex, BMI, MAP, eGFR, Oxford MEST-C score, use of RAS inhibitiors, and immunosuppression during follow-up.

We created three prediction models to evaluate whether incorporating biomarkers levels will improve the predictive ability. Univariate models included only the biomarkers (u-sCD163/Cr, u-IL6/Cr or u-MCP-1/Cr). Clinical models included major clinical risk factors (MAP, 24-hour proteinuria, and eGFR) at time of biopsy and MEST-C component scores. Predictive power was quantified through the area under the receiver operating characteristic (AUROC) curves. Delong test was used to compare the significant differences between ROC curves.

## Results

### Characteristics of Patients Grouped by u-sCD163

From January 2017 to December 2018, 349 eligible (191 men and 158 women) IgAN patients with an average age of 40.95 ± 12.73 years were recorded in the database. We summarized patient characteristics, clinical data, and kidney histological evaluations based on u-sCD163/Cr levels (**Table 1**). The mean MAP at baseline was 98.83 ± 11.84 mmHg. Mean eGFR was 74.35 ± 29.35 mL/min/1.73m^2^, and mean proteinuria was 1.47 ± 1.22 g/24 h (**Figure 1** and **Table 1**). The 262 patients who were followed up for 6 months had comparable baseline conditions as those not followed up (**Table S1**).

**Table 1:** Characteristics of IgAN patients by urinary CD163 tertiles at biopsy.

		Urinary sCD163 (ng/mg Cr)		
Variable^a^	Overall	T1 (<1.27)	T2 (1.27-3.57)	T3 (＞3.57)	*P* ^b^
No. of patients	349	116	117	116	–
Age, y	40.95 ± 12.73	40.59 ± 12.47	39.45 ± 12.01	42.80 ± 13.54	0.125
Male	191 (54.7%)	78 (67.2%)	63 (53.8%)	50 (43.1%)	0.001
BMI, kg/m^2^	24.33 ± 3.63	24.65 ± 3.30	24.01 ± 3.79	24.32 ± 3.79	0.409
Hypertension	178 (51.0%)	58 (50.0%)	58 (49.6%)	62 (53.4%)	0.811
Diabetes	17 (4.9%)	7 (6%)	3 (2.6%)	7 (6%)	0.364
MAP, mmHg	98.83 ± 11.84	97.03 ± 10.72	99.65 ± 12.18	99.82 ± 12.42	0.131
Hemoglobin, g/L	128.55 ± 19.46	134.82 ± 17.40	129.32 ± 20.05	121.63 ± 19.68	<0.0001
Serum albumin, g/L	39.04 ± 4.74	41.03 ± 3.44	39.79 ± 4.08	36.33 ± 5.24	<0.0001
CRP, mg/dL	0.20 ± 0.38	0.17 ± 0.34	0.21 ± 0.41	0.23 ± 0.37	0.437
Serum creatinine, mg/dL	1.28 ± 0.65	1.13 ± 0.40	1.27 ± 0.60	1.43 ± 0.83	0.002
eGFR, mL/min/1.73m^2^	74.35 ± 29.35	81.97 ± 25.56	74.51 ± 28.36	66.59 ± 31.98	<0.0001
Proteinuria, g/24h	1.47 ± 1.22	0.75 ± 0.63	1.33 ± 0.96	2.33 ± 1.36	<0.0001
Urinary IL6, ng/mg Cr	54.38 ± 65.54	30.10 ± 28.27	51.94 ± 63.41	80.91 ± 82.51	<0.0001
Urinary MCP-1, ng/mg Cr	561.90 ± 501.73	331.60 ± 316.51	508.65 ± 368.80	843.92 ± 622.03	<0.0001
Use ACEI/ARBs at biopsy	78 (22.3%)	26 (22.4%)	30 (25.6%)	22 (19%)	0.473
Renal biopsy Lee’s classification					<0.0001
Mild (I-II)	49 (14.0%)	32 (27.6%)	12 (10.3%)	5 (4.3%)	
Moderate (III)	79 (22.6%)	34 (29.3%)	30 (25.6%)	15 (12.9%)	
Severe (IV-V)	221 (63.3%)	50 (43.1%)	75 (64.1%)	96 (82.8%)	
Oxford MEST-C					
M1	302 (86.5%)	78 (67.2%)	111 (94.9%)	113 (97.4%)	<0.0001
E1	46 (13.2%)	7 (6.0%)	15 (12.8%)	24 (20.7%)	0.004
S1	191 (54.7%)	44 (37.9%)	66 (56.4%)	81 (69.8%)	<0.0001
T1-2	156 (44.7%)	30 (25.9%)	53 (45.3%)	73 (62.9%)	<0.0001
C1-2	113 (38.1%)	17 (14.7%)	49 (41.9%)	67 (57.8%)	<0.0001

BMI, body mass index; eGFR, estimated glomerular filtration rate; MAP, mean arterial blood pressure; CRP, C-reactive protein; U-MCP-1, urinary monocyte chemoattractant protein-1; ACEI, Angiotensin-converting enzyme inhibitors; ARBs, Angiotensin II receptor blockers; MEST-C, histologic score based on mesangial hypercellularity, the presence of endocapillary proliferation, segmental glomerulosclerosis/adhesion, and severity of tubular atrophy/interstitial fibrosis, and crescents formation; T, tertile.

a Continuous variables are expressed as mean ± standard deviation. Categorial variables are expressed as number (percent).

b Comparing the covariated across the 3 urinary sCD163 categories.

### Correlation Between u-sCD163 and Clinical Parameters of the IgAN Patients

**Figure 2:**
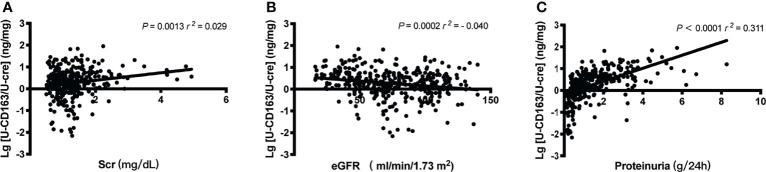
Urinary sCD163 (u-sCD163) is associated with proteinuria and eGFR in IgAN patients. **(A)** Correlation between u-sCD163 levels and serum creatine. **(B)** Correlation between U-sCD163 levels and eGFR. **(C)** Correlation between U-sCD163 levels and 24-hour proteinuria; each dot represents a value from an individual patient. Coefficients of determination (*r* ^2^) and *P*-values are shown.

We first explore the relationship between u-sCD163 levels with clinical index. Levels of u-sCD163 were positively correlated with serum creatine concentration (*r* = 0.029, *p*<0.05; **Figure 2A**) and negatively correlated with eGFR at the time of renal biopsy (*r* = -0.040, *p*<0.001; **Figure 2B**). Proteinuria was also a feature of IgAN. Urinary sCD163 was positively correlated with the levels of 24-hour proteinuria (*r* = 0.311, *p*<0.001; **Figure 2C**). The high u-sCD163 group had significantly lower hemoglobin and serum albumin levels (T3, *p*<0.0001; **Table 1**). Together, these results indicate that elevated u-sCD163 is associated with severe clinical manifestations.

### Association Between u-sCD163 Levels and Renal CD163^+^ Cells Infiltration

**Figure 3:**
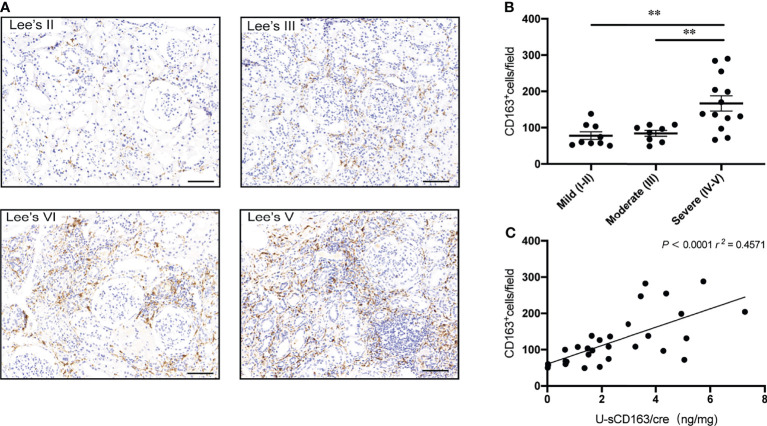
Urinary sCD163 levels are correlated with tubulointerstitial CD163^+^ macrophage infiltration. **(A)** CD163^+^ macrophages in IgAN classified according to Lee’s pathological grade. Bar: 50 µm. **(B)** Quantitative analysis of CD163^+^ cell counts in tubulointerstitial lesions from IgAN patients assigned different Lee’s pathological grades. **P<0.001. **(C)** Correlation between tubulointerstitial CD163^+^ cell count and u-sCD163 levels; each dot represents a value from an individual patient and the group mean is shown as a horizontal bar. Coefficients of determination (*r* ^2^) and *P*-values are shown.

In patients with LN and ANCA-GN patients, u-sCD163 is correlated with glomerular CD163^+^ macrophages (12, 14). However, in our IgAN patients, CD163^+^ macrophages were mainly expressed in tubulointerstitial lesions, and tubulointerstitial CD163^+^ cells were significantly higher in Lee’s pathological grade IV-V patients (**Figures 3A, B**). Urinary sCD163 levels were highly correlated with tubulointerstitial CD163^+^ macrophages (*r* = 0.457, *p*<0.0001; **Figure 3C**). Our IgAN cohort did not exhibit a significant correlation between u-sCD163 levels and glomerular CD163^+^ macrophages (data not shown).

### Urinary sCD163 and Histology

**Figure 4:**
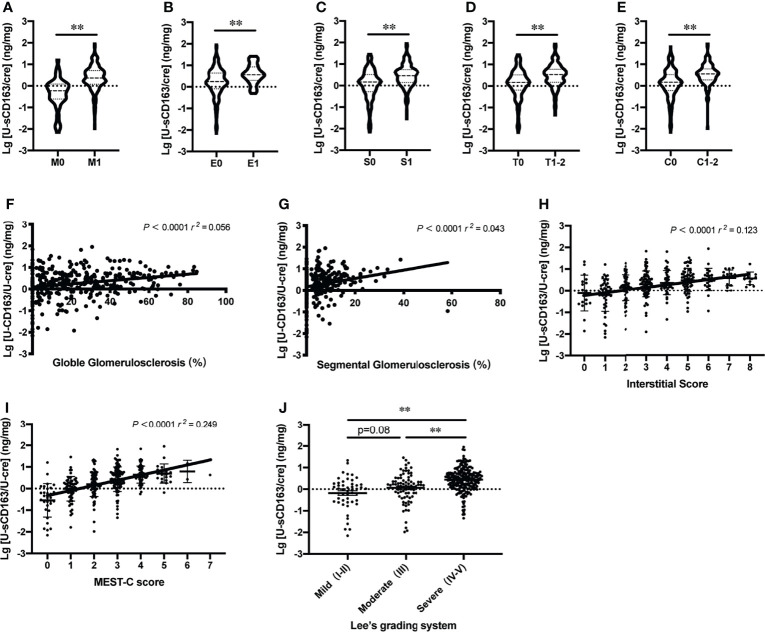
Urinary sCD163 levels reflect patient histology. **(A–E)** Association between u-sCD163 levels in IgAN patients with Oxford Classification. **P<0.001. **(F)** Correlation between u-sCD163 levels and global glomerulosclerosis. **(G)** Correlation between u-sCD163 levels and segmental glomerulosclerosis. **(H)** Correlation between u-sCD163 levels and interstitial score. **(I)** Correlation between u-sCD163 levels and MEST-C score; each dot represents a value from an individual patient Coefficients of determination (*r* ^2^) and *P*-values are shown. **(J)** Quantitative analysis of u-sCD163 levels in IgAN patients with differing Lee’s pathological grades. **P<0.001.

To confirm whether u-sCD163 is a potential biomarker for disease activity assessment, we analyzed the correlation between u-sCD163 concentration and histology in kidney specimens from patients with IgAN. We first analyzed the association between u-sCD163 levels and the Oxford Classification. Our univariate analysis determined that patients with mesangial hypercellularity and those with endocapillary hypercellularity both had higher u-sCD163 levels (*p* < 0.0001 and *p* = 0.0002, respectively; **Figures 4A, B**). Urinary sCD163 levels increased in patients presenting segmental glomerulosclerosis (*p*<0.0001; **Figure 4C**). Additionally, u-sCD163 levels were significantly correlated with global glomerulosclerosis score (*r* = 0.056, *p*<0.0001; **Figure 4F**) and segmental glomerulosclerosis score (*r* = 0.043, *p*<0.0001; **Figure 4G**). Urinary sCD163 increased in tubulointerstitial damage group (T1-2, *p*<0.0001; **Figure 4D**), and was highly correlated with tubulointerstitial score (*r* = 0.123, *p*<0.0001; **Figure 4H**). Patients with > 25% renal crescent proportion had higher u-sCD163 levels (*p*<0.0001; **Figure 4E**). Furthermore, u-sCD163 was correlated with MEST-C score (*r* = 0.249, *p*<0.0001; **Figure 4I**), and patients with Lee’s pathological grade IV-V had significantly higher u-sCD163 (*p*<0.0001; **Figure 4J**). Biomarkers u-IL6 and u-MCP-1were both significantly higher in Lee’s pathological grade IV-V group, as well (*p*<0.0001; **Supplementary Figures 1A, B**). Taken together, these results indicated that u-sCD163 levels reflect the histology states and may serve as a noninvasive biomarker of IgAN.

### Association Between Urinary Biomarkers and IgAN Remission Status

Sustained proteinuria >1 g/24 h was the strongest predictor of renal disease progression rate and renal failure in IgAN (19). Therefore, we analyzed the relationship with urinary biomarkers and IgAN remission status. Compared with u-IL6 and u-MCP-1, u-sCD163 had the strongest association with IgAN remission status. In particular, the association with u-MCP-1 was no longer significant after adjustments in Model 3 (age, sex, MAP, BMI, 24-hour proteinuria, eGFR, Oxford MEST-C score, and use of renin-angiotensin system inhibition or immunosuppression during follow-up). By contrast, Model 3 showed that patients in the highest u-sCD163 tertile (>3.57 ng/mg Cr) at biopsy had a 2.66-fold greater risk for IgAN remission failure than those in the lower two tertiles combined (≤3.57 ng/mg Cr; **Table 2**). Thus, u-sCD163 concentrations greater than the spline cutoff value (3.57 ng/mg Cr) were independently associated with IgAN remission status. Furthermore, u-sCD163 level was independent predictor of IgAN remission status after stepwise backward selection. (**Supplementary Table 2**). **Supplementary Table 3** summarizes profiles, clinical data, and kidney histological evaluations based on remission status of the 262 patients who were followed up.

**Table 2:** Multivariable logistic analyses of urinary biomarkers for predicting IgAN remission status.

	Cut Pointsng/mg Cr	Remission failure %	Unadjusted OR (95% Cl); *P*	Adjusted OR (95% Cl)		
				Model 1^a^, *P*	Model 2^b^, *P*	Model 3^c^, *P*
Urinary sCD163						
T1+T2 (n=175)	≤3.57	14.3	1.0 (referent)	1.0 (referent)	1.0 (referent)	1.0 (referent)
T3 (n=87)	>3.57	37.9	3.67 (2.00-6.72); <0.0001	3.91 (2.06-7.41); <0.0001	2.32 (1.09-4.90); 0.028	2.66 (1.22-5.86); 0.014
Urinary IL6						
T1+T2 (n=175)	≤52.36	17.1	1.0 (referent)	1.0 (referent)	1.0 (referent)	1.0 (referent)
T3 (n=87)	>52.36	32.6	2.28 (1.26-4.12); 0.007	2.33 (1.25-4.37); 0.008	1.99 (1.03-3.88); 0.042	2.06 (1.05-4.03); 0.035
Urinary MCP-1						
T1+T2 (n=175)	≤542.48	17.2	1.0 (referent)	1.0 (referent)	1.0 (referent)	1.0 (referent)
T3 (n=87)	>542.48	32.2	2.28 (1.26-4.12); 0.007	2.49 (1.31-4.73); 0.006	1.69 (0.84-3.38); 0.140	1.72 (0.85-3.47); 0.130

Cl, confidence interval; Cr, creatinine; OR, Odds ratio; MCP-1, monocyte chemoattractant protein-1; BMI, body mass index; eGFR, estimated glomerular filtration rate; MAP, mean arterial blood pressure; MEST-C, histologic score based on mesangial hypercellularity, the presence of endocapillary proliferation, segmental glomerulosclerosis/adhesion, and severity of tubular atrophy/interstitial fibrosis, and crescents formation; T, tertile.

a Model 1 adjusted for age, sex, MAP, BMI.

b Model 2 adjusted for covariates in model 1 plus 24-hour proteinuria, eGFR and Oxford MEST-C score.

c Model 3 adjusted for covariates in model 1 and 2 plus use of renin-angiotensin system inhibition and immunosuppression during follow-up.

### Use of u-sCD163 Levels With Clinical and Histologic Data for Predicting IgAN Remission Status

We first used univariable models to analyze the prediction performance of urinary biomarkers (**Table 3**). Urinary sCD163 (AUC 0.732, 95% CI, 0.658-0.807) had much stronger predictive power than u-IL6 (AUC 0.661, 95% CI, 0.586-0.735) and u-MCP-1(AUC 0.638, 95% CI, 0.560-0.716). Adding u-sCD163 levels to the model containing clinical data at biopsy (MABP, 24-hour proteinuria, and eGFR) and MEST-C score significantly improved the prediction of IgAN remission status (AUC 0.788, 95% CI, 0.717-0.860) over the univariate model (Delong test, p = 0.05). However, adding other biomarkers to the model containing u-sCD163, clinical data, and MEST-C did not further improve predictive performance (AUC 0.784, 95% CI, 0.711 - 0.858; Delong test, p = 0.77; **Table 3**).

**Table 3:** Performance of biomarkers and/or clinical data for prediction IgAN remission status.

	AUC (95% Cl) ^a^ (n=262)
Univariable models of biomarkers	
Urinary sCD163 at biopsy	0.732 (0.658-0.807)
Urinary IL6 at biopsy	0.661 (0.586-0.735)
Urinary MCP-1 at biopsy	0.638 (0.560-0.716)
Clinical models	
Clinical data at biopsy^b^	0.768 (0.688-0.849)
Clinical data at biopsy + MEST-C^c^	0.769 (0.690-0.848)
Models containing clinical data and biomarkers	
Clinical data at biopsy + MEST-C + urinary sCD163	0.788 (0.717-0.860)
Clinical data at biopsy + MEST-C + urinary IL6	0.774 (0.697-0.851)
Clinical data at biopsy + MEST-C + urinary MCP-1	0.778 (0.704-0.851)
Models containing clinical data and multiple biomarkers	
Clinical data at biopsy + MEST-C + urinary sCD163 + IL6 + MCP-1	0.784 (0.711-0.858)

AUC, Area under the Curve of ROC; Cl, confidence interval; MCP-1, monocyte chemoattractant protein-1; MEST-C, histologic score based on mesangial hypercellularity, the presence of endocapillary proliferation, segmental glomerulosclerosis/adhesion, and severity of tubular atrophy/interstitial fibrosis, and crescents formation.

a Expressed as AUC, continuous biomarkers were used in the model.

b Comprising mean arterial blood pressure, 24 hour proteinuria, and estimated glomerular filtration rate at biopsy.

c Oxford histologic classification.

## Discussion

To the best of our knowledge, this study is the first to demonstrate that urinary sCD163, measured at the time of kidney biopsy, could be a reliable noninvasive biomarker for monitoring renal injury in patients with IgAN. We further demonstrated that u-sCD163 levels are independent and strong predictor for IgAN remission status.

This is also the first study to investigate how u-sCD163 relates to histological and clinical characteristics of IgAN. We found that elevated u-sCD163 is correlated with severe clinical manifestations. It was positively associated with 24-hour proteinuria excretion and serum creatinine, but negatively associated with eGFR, hemoglobin, and serum albumin. We also analyzed u-sCD163 concentration across different histopathological changes and found it to be correlated with MEST-C score, as well as being significantly higher in Lee’s grades IV-V than in grades I-II and III. These findings together suggest that u-sCD163 is closely associated with IgAN clinical severity and histologic activity. Patients may exhibit higher levels of u-sCD163 levels if their kidney damage is more severe.

Another key finding from this study was that u-sCD163 levels were highly correlated with tubulointerstitial CD163^+^ macrophages. SCD163 derives from cleavage of the CD163^+^ macrophage receptor (20). CD163^+^ macrophages are considered to be M2c macrophages with anti-inflammatory and pro-fibrotic functions (10). Indeed, previous studies on IgAN showed that CD163^+^ macrophage subsets correlated with degree of interstitial fibrosis (21), and that CD163^+^ macrophages play a more important role in progression of IgAN fibrosis than other kinds of macrophages (8). Relatedly, we demonstrated that u-sCD163 levels were higher in patients with severe tubule-interstitial lesions, while being significantly correlated with global glomerulosclerosis and segmental glomerulosclerosis score. *In vitro* experiments indicated that incubation of human renal proximal tubular epithelial cells (HK-2) with M2c macrophages promoted epithelial-to mesenchymal transition (EMT) of HK-2 cells (22). In a single-cell atlas of mouse model of unilateral ureteral obstruction (UUO), monocytes recruited to the kidney early after injury rapidly adopt a proinflammatory, profibrotic phenotype that expresses Arg1^+^ M2 macrophages (23). In murine adriamycin nephrosis, transferred M2c macrophages effectively reduced glomerulosclerosis tubular atrophy, interstitial expansion and proteinuria (24). While these animal studies suggest a major role of macrophages in renal disease, developing animal models for IgAN is a major challenge. Therefore, we currently do not have a clear understanding of macrophage function in IgAN, and the topic requires further investigation.

In normal population, u-sCD163 levels are very low (12). Urinary sCD163 is also elevated in several active glomerular diseases. In LN and ANCA-GN, for example, u-sCD163 behaves as a histologic biomarker that correlates with the number of CD163^+^ cells infiltrating the glomeruli (12–14). However, in this study, we didn’t find a significant relationship between u-sCD163 levels and glomerular CD163^+^ macrophages among our IgAN cohort, possibly because glomerular lesions in IgAN are less severe than those in LN and ANCA-GN. Additionally, more macrophages accumulate in tubulointerstitial lesions of IgAN, instead of glomerular legions.

We also found that patients with >25% renal crescent proportion have higher u-sCD163 levels. A multi-center study included 113 Chinese patients with crescentic IgAN reported poor prognosis; nearly 70% of these patients progressed to ESKD in 5 years (25). Therefore, we suggest that high u-sCD163 levels reflect active lesions, especially IgAN crescents.

Proteinuria is an adverse prognostic factor in IgAN (19). A large, international, and multi-center study reported that among IgAN patients who achieved proteinuria remission (reduction in proteinuria to an absolute value < 1 g/24 h), every 3 months in remission was associated with an additional 9% reduction in the risk of ESKD, or a 50% decline in kidney function over a median follow-up of 3.9 years (26). Therefore, short periods of proteinuria remission are associated with lowered risk of IgAN progression. Here we defined short-term RF either as proteinuria > 1 g/24 h or as one of the following symptoms: increase in proteinuria, renal deterioration (≥30% eGFR decline), or ESKD 6 months after renal biopsy. Patients with a base line u-sCD163 levels > 3.57 ng/mg Cr had a 2.66-fold higher risk for short-term RF than those with those with u-sCD163 levels ≤ 3.57 ng/mg Cr after adjusted by age, sex, MAP, BMI 24-hour proteinuria, eGFR, Oxford MEST-C score, and use of RAS inhibition and immunosuppression during follow-up.

Of note, there are few reliable markers for assessing the inflammatory activity of the monocyte/macrophage lineage in IgAN. Both MCP-1 and IL-6 are produced by monocytes/macrophages, neutrophils, endothelial cells, and epithelial cells. The two proteins are important to chronic inflammation in IgAN, and their urinary levels are the most potent biomarkers for the disease to date (15, 27). However, we found that their predictive performance was weaker than u-sCD163. U-sCD163 reflects the proinflammatory status in IgAN. Our multivariable analyses revealed that u-sCD163 was a strong predictor of IgAN short-term RF after adjusting for clinical traits, histological features, and subsequent treatment. Combining u-sCD163 levels, clinical data, and MEST-C score in one model significantly improved prediction of IgAN remission status. Urinary sCD163 levels delivered better predictive performance than the other two biomarkers in both uni- and multivariate models.

These data showing improved risk prediction at the time of diagnosis suggests that u-sCD163 should become very valuable for treatment decisions. Another advantage is that sCD163 is stable in urine for at least 1 week at room temperature (28). Thus, variations in samples will not be a determining factor in the assay result. Even in remote hospitals where kidney biopsies cannot be performed due to limited resources, urine samples can be collected and delivered to a testing center. Urinary sCD163 is a promising biomarker that may drastically change clinical practices in developing regions where kidney biopsies are uncommon.

This study had several limitations. First, all participating patients were from a single center, meaning our results require validation in other independent IgAN cohorts before any clinical application. Second, only baseline u-sCD163 levels were assessed, so further evaluations on variation over time and treatment response are needed. Finally, we did not examine associations of u-sCD163 with long-term renal outcomes of IgAN patients because renal replacement therapy and death were limited during our observation period.

In conclusion, higher u-sCD163 levels were associated with increased severity in histological lesions, as well as elevated proteinuria and poorer renal function among IgAN patients. We successfully demonstrated that u-sCD163 is an independent and strong predictor for IgAN remission status, suggesting that it is a candidate non-invasive biomarker of the disease.

## Data Availability Statement

The raw data supporting the conclusions of this article will be made available by the authors, without undue reservation.

## Ethics Statement

The studies involving human participants were reviewed and approved by The Ethics Committee of Zhongshan Hospital. The patients/participants provided their written informed consent to participate in this study.

## Author Contributions

All authors listed have made a substantial, direct and intellectual contribution to the work, and approved it for publication.

## Funding

This research was funded by the National Natural Science Foundation of China and the Science 81870476, 81803880, 81800592, 82103911; Shanghai ‘Rising Stars of Medical Talent’ Youth Development Program (YS); Shanghai Sailing Program (18YF1419800); Clinical and Experimental Research for YSHS Granule (XX); Shanghai Key Laboratory of Kidney and Blood Purification (14DZ2260200, 20DZ2271600); Shanghai Medical Centre of Kidney (2017ZZ01015).

## Conflict of Interest

The authors declare that the research was conducted in the absence of any commercial or financial relationships that could be construed as a potential conflict of interest.

## Publisher’s Note

All claims expressed in this article are solely those of the authors and do not necessarily represent those of their affiliated organizations, or those of the publisher, the editors and the reviewers. Any product that may be evaluated in this article, or claim that may be made by its manufacturer, is not guaranteed or endorsed by the publisher.

## Supplementary Material

Supplementary Figure 1 U-IL6 and U-MCP1 levels reflect the histology status. **(A)** Quantitative analysis of u-IL6 levels in IgAN patients with different Lee’s pathological grade. **(B)** Quantitative analysis of u-MCP1 levels in IgAN patients with different Lee’s pathological grade. *P < 0.05, **P < 0.001.
