# Supplementary figures and images for: Urinary Soluble CD163 Levels Predict IgA Nephropathy Remission Status
Source: Front Immunol. 2021 Dec 23;12:769802. doi: 10.3389/fimmu.2021.769802 (PMC8733336; doi:10.3389/fimmu.2021.769802)

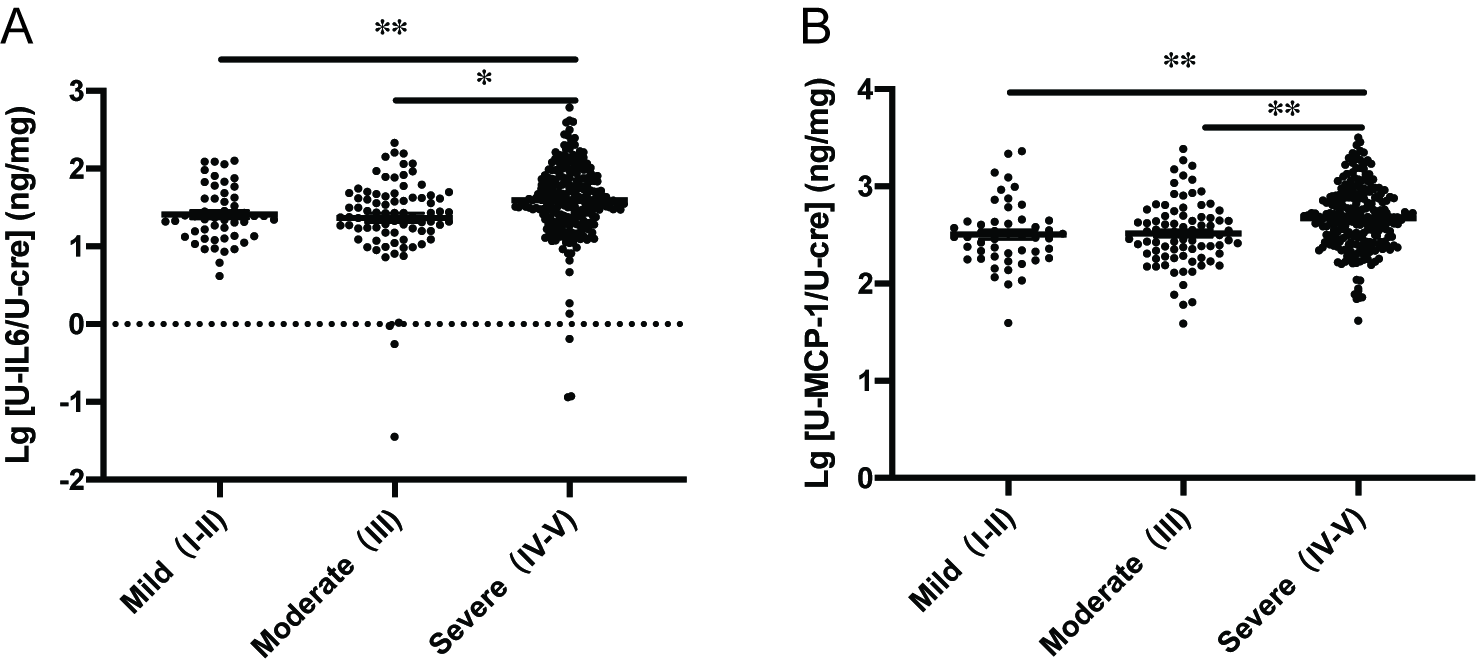

Supplement: Supplementary Figure 1 — U-IL6 and U-MCP1 levels reflect the histology status. (A) Quantitative analysis of u-IL6 levels in IgAN patients with different Lee’s pathological grade. (B) Quantitative analysis of u-MCP1 levels in IgAN patients with different Lee’s pathological grade. *P < 0.05, **P < 0.001. [file Image_1.tif]
