# Supplementary material for: Urinary Soluble CD163 Levels Predict IgA Nephropathy Remission Status
Source: Front Immunol. 2021 Dec 23;12:769802. doi: 10.3389/fimmu.2021.769802 (PMC8733336; doi:10.3389/fimmu.2021.769802)
Supplement: Supplementary file 2 [file DataSheet_1.docx]

| **Variable^a^** | **Followed-up** | **Unfollowed-up** | ***P* ^b^** |
| --- | --- | --- | --- |
| No. of patients | 262 | 87 | - |
| Age, y | 41.27±12.80 | 39.97±12.54 | 0.408 |
| Male | 144 (55.0%) | 48 (55.2%) | 0.923 |
| BMI, kg/m^2^ | 24.29±3.48 | 24.43±4.07 | 0.765 |
| Hypertension | 138 (52.7%) | 40 (46.0%) | 0.279 |
| MAP, mmHg | 99.51±12.02 | 96.78±11.08 | 0.062 |
| Hemoglobin, g/L | 128.86±19.83 | 127.6±18.37 | 0.605 |
| Serum albumin, g/L | 38.85±4.84 | 39.64±4.40 | 0.185 |
| CRP, mg/dL | 2.00±3.78 | 2.02±3.71 | 0.968 |
| Serum creatinine, mg/dL | 1.28±0.62 | 1.26±0.73 | 0.747 |
| eGFR, mL/min/1.73m^2^ | 72.87±28.13 | 78.83±32.51 | 0.101 |
| Proteinuria, g/24h | 1.47±1.16 | 1.45±1.39 | 0.876 |
| U-CD163, ng/mg Cr | 4.61±7.76 | 4.23±10.24 | 0.722 |
| U-IL6, ng/mg Cr | 54.97±56.49 | 55.60±87.64 | 0.841 |
| U-MCP-1, ng/mg Cr | 560.70±476.91 | 565.48±572.77 | 0.939 |
| Renal biopsy Lee’s classification | |  | 0.209 |
| Mild (I-II) | 32 (12.2%) | 17 (19.5%) |  |
| Moderate (III) | 59 (22.5%) | 20 (23.0%) |  |
| Severe (IV-V) | 171 (65.3%) | 50 (57.5%) |  |
| Oxford MEST-C |  |  |  |
| M1 | 231 (88.2%) | 71 (81.6%) | 0.120 |
| E1 | 37 (14.1%) | 9 (10.3%) | 0.367 |
| S1 | 147 (56.1%) | 44 (50.6%) | 0.369 |
| T1-2 | 125 (47.7%) | 31 (35.6%) | 0.062 |
| C1-2 | 107 (40.8%) | 26 (29.9%) | 0.068 |
| Abbreviations: BMI, body mass index; eGFR, estimated glomerular filtration rate; MAP, mean arterial blood pressure; CRP, C-reactive protein; U-MCP-1, urinary monocyte chemoattractant protein-1; MEST-C, histologic score based on mesangial hypercellularity, the presence of endocapillary proliferation, segmental glomerulosclerosis/adhesion, and severity of tubular atrophy/interstitial fibrosis, and crescents formation; T, tertile.  ^a^Continuous variables are expressed as mean ± standard deviation. Categorial variables are expressed as number (percent).  ^b^Comparing the covariated between followed-up and unfollowed-up. | | | |

**Supplement Table1. Characteristics of IgAN Patients by followed-up or not**

**Supplement Table2. Stepwise Multiple Logistic Regression for Variables Associated with IgAN Remission Status**

| **Variable** | **Compare Group** | **Control Group** | **OR （95% CI）** | ***P*** |
| --- | --- | --- | --- | --- |
| Urinary sCD163 | T3 | T1+T2 | 3.233 (1.594-6.558) | 0.001 |
| BMI, kg/m^2^ | Continuous variable | | 1.084 (0.988-1.190) | 0.088 |
| MEST-C | Continuous variable | | 1.384 (1.006-1.903) | 0.046 |
| eGFR, mL/min/1.73m^2^ | Continuous variable | | 0.984 (0.972-0.997) | 0.015 |
| Use immunosuppression during follow-up | Yes | No | 0.510 (0.230-1.130) | 0.097 |
| Abbreviations: OR, Odds ratio; Cl, confidence interval; Cr, creatinine; OR, Odds ratio; MCP-1, monocyte chemoattractant protein-1; BMI, body mass index; MEST-C, histologic score based on mesangial hypercellularity, the presence of endocapillary proliferation, segmental glomerulosclerosis/adhesion, and severity of tubular atrophy/interstitial fibrosis, and crescents formation; eGFR, estimated glomerular filtration rate. | | | | |

**Supplement Table3. Characteristics of IgAN Patients by Remission Status**

| **Variable^a^** | **Overall** | **CR** | | **PR** | **RF** | ***P* ^b^** |
| --- | --- | --- | --- | --- | --- | --- |
| No. of patients | 262 | 87 | | 117 | 58 | - |
| Age, y | 41.27±12.80 | 40.11±10.77 | | 41.01±13.51 | 43.53±14.01 | 0.277 |
| Male | 191 (54.7%) | 49 (56.3%) | | 63 (53.8%) | 31 (53.4%) | 0.922 |
| BMI, kg/m^2^ | 24.29±3.48 | 23.91±3.42 | | 24.34±3.28 | 24.78±3.94 | 0.336 |
| Hypertension | 138 (52.7%) | 37 (42.5%) | | 62 (53.0%) | 39 (67.2%) | 0.014 |
| MAP, mmHg | 98.51±12.02 | 96.68±12.46 | | 101.18±11.89 | 100.41±11.00 | 0.024 |
| Hemoglobin, g/L | 128.86±19.83 | 130.09±19.10 | | 130.82±20.81 | 123.05±18.00 | 0.041 |
| Serum albumin, g/L | 38.85±4.84 | 40.15±5.04 | | 39.13±3.97 | 36.29±5.27 | ＜0.0001 |
| CRP, mg/dL | 0.20±0.38 | 0.17±0.31 | | 0.20±0.37 | 0.25±0.47 | 0.438 |
| Serum creatinine, mg/dL | 1.28±0.62 | 1.13±0.37 | | 1.19±0.40 | 1.70±1.00 | ＜0.0001 |
| eGFR, mL/min/1.73m^2^ | 72.87±28.13 | 79.36±24.91 | | 74.67±25.22 | 59.49±33.78 | ＜0.0001 |
| Proteinuria, g/24h | 1.47±1.22 | 0.96±1.07 | | 1.43±0.86 | 2.32±1.33 | ＜0.0001 |
| Urinary sCD163, ng/mg Cr | 4.61±7.76 | 2.47±3.64 | | 3.58±4.07 | 9.88±13.58 | ＜0.0001 |
| Urinary IL6, ng/mg Cr | 53.97±56.49 | 42.74±39.39 | | 54.72±62.93 | 69.12±61.30 | 0.022 |
| Urinary MCP-1, ng/mg Cr | 560.70±476.91 | 483.97±430.42 | | 537.46±424.23 | 721.38±598.89 | 0.010 |
| Renal biopsy Lee’s classification | | |  | | | 0.038 |
| Mild (I-II) | 32 (12.2%) | 16 (18.4%) | | 13 (11.1%) | 3 (5.2%) |  |
| Moderate (III) | 59 (22.5%) | 23 (26.4%) | | 27 (23.1%) | 9 (15.5%) |  |
| Severe (IV-V) | 171 (65.3%) | 48 (55.2%) | | 77 (65.8%) | 46 (79.3%) |  |
| Oxford MEST-C |  |  | |  |  |  |
| M1 | 231 (88.2%) | 67 (77.0%) | | 109 (93.2%) | 55 (94.8%) | ＜0.0001 |
| E1 | 37 (14.1%) | 11 (12.6%) | | 14 (12.0%) | 12 (20.7%) | 0.263 |
| S1 | 147 (56.1%) | 41 (47.1%) | | 67 (57.3%) | 39 (67.2%) | 0.054 |
| T1-2 | 125 (47.7%) | 30 (34.5%) | | 55 (47.0%) | 40 (69.0%) | ＜0.0001 |
| C1-2 | 107 (40.8%) | 30 (34.5%) | | 51 (43.6%) | 26 (44.8%) | 0.332 |
| Abbreviations: CR, complete remission; PR, partial remission; RF, remission failure; BMI, body mass index; eGFR, estimated glomerular filtration rate; MAP, mean arterial blood pressure; CRP, C-reactive protein; U-MCP-1, urinary monocyte chemoattractant protein-1; MEST-C, histologic score based on mesangial hypercellularity, the presence of endocapillary proliferation, segmental glomerulosclerosis/adhesion, and severity of tubular atrophy/interstitial fibrosis, and crescents formation; T, tertile.  ^a^Continuous variables are expressed as mean ± standard deviation. Categorial variables are expressed as number (percent).  ^b^Comparing the covariated across 3 remission status (CR, PR and RF). | | | | | | |
